# Antibacterial activity of *Centaurea pumilio* L. root and aerial part extracts against some multidrug resistant bacteria

**DOI:** 10.1186/s12906-020-2876-y

**Published:** 2020-03-12

**Authors:** Huda Naeim, Amr El-Hawiet, Raoufa A. Abdel Rahman, Ahmed Hussein, Maha A. El Demellawy, Amira M. Embaby

**Affiliations:** 1 Department of Biotechnology, Institute of Graduate Studies and Research, Alexandria University, 163 Horreya Avenue, Chatbye, P.O.Box 832, Alexandria, Egypt; 2 Pharmaceutical Bioproducts Research Department, Genetic Engineering & Biotechnology Research Institute (GEBRI), City of Scientific Research and Technological Applications (SRTA-City), New Borg El-Arab City, Alexandria, Egypt; 3 Medical Biotechnology Department, GEBRI, SRTA-City, New Borg El-Arab City, Alexandria, Egypt; 4 Department of Pharmacognosy, Faculty of Pharmacy, Alexandria University, Alexandria, Egypt

**Keywords:** AMR, *Centaurea pumilio* L*.*, Aerial part, Root, Essential oil, Plant extracts, Antimicrobial activity, MDR strains, MIC, GC-MS

## Abstract

**Background:**

In the context of searching for potent, safe, natural antimicrobial agents to combate the global antimicrobial resistance (AMR) phenomenon, the current study evaluates for the first time ever, the broad-spectrum antimicrobial activity of essential oil (EO) and extracts from the rare wild plant *Centaurea pumilio* L*.*. It has tremendous ethnomedicinal values; its dried root is used as a fattening agent, a treatment for bad breath and diabetes, and screened for schistosomicidal activity.

**Methods:**

*C. pumilio* EO was extracted by hydrodistillation using a Clevenger apparatus. Chemical constituents of aerial part were extracted using a sequential solvent/solvent procedure employing four solvents with increasing polarities in the following order: petroleum ether, chloroform, ethyl acetate, and *n*-butanol. The chemical constituents were identified by GC-MS. Fifty-two microbial strains were used; twenty-six multidrug resistant (MDR), sixteen clinical, and ten reference strains. The identification of the microbial strains was performed by MALDI-TOF-MS. The antimicrobial activity of the EO and the aerial part and the root extracts was assessed through disc diffusion assay. A minimum inhibitory concentration (MIC) of the EO and extracts was determined using the broth micro-dilution method.

**Results:**

The growth of reference and clinical strains was inhibited by EO, methanol, chloroform, and ethyl acetate aerial part extracts and chloroform root extract. The MDR strains growth, however, was inhibited only by EO and chloroform aerial part extract*.* GC-MS identified for the first time eighteen constituents from aerial part EO and chloroform extract each. EO showed antimicrobial activity against the reference, clinical, and MDR strains with MIC values of 31.25–125, 31.25–125, and 62.50–250 μg/mL, respectively. Methanol aerial part extract exhibited high antimicrobial activities with MIC values of 62.50–250 μg/mL against reference and clinical strains. Chloroform root extract displayed strong antimicrobial activity against reference and clinical strains recording MIC values of 62.50–250 μg/mL and 62.50–125 μg/mL, respectively. The chloroform aerial part extract demonstrated potent antimicrobial activity against the reference, clinical, and MDR strains with 31.25, 31.25, and 15.62 μg/mL MIC values, respectively.

**Conclusions:**

Present data unravel the *C. pumilio* pharmacological magnitude to discover eco-friendly potent antimicrobial agents to fight AMR phenomenon.

## Background

Antimicrobial resistance (AMR), a life-threatening and multifaceted global phenomenon, is a consequence of improper and/or overuse of antibiotics [1]. The Centre for Disease Control and Prevention (CDC) and the World Health Organization (WHO) report alarmingly increasing mortality rates as a result of infections from various MDR strains [2]. Some of the most life-threatening MDR strains with severe human implications worldwide are *Staphylococcus aureus* MRSA, *Acinetobacter baumannii*, *Pseudomonas aeruginosa*, *Escherichia coli*, and *Klebsiella pneumonia* [3]. Globally, intensive care units (ICUs) are considered foci for the proliferation and promotion of persistent infection with MDR strains [4]. In Egypt, several studies have been conducted to trace the incidence and prevalence of AMR and its possible reasons, especially in ICUs [5, 6]. Infection with Gram-negative MDR strains (e.g., *Acinetobacter* sp*., Klebsiella* sp*.*, and *Pseudomonas* sp*.*) with a higher frequency than Gram-positive MDR strains was reported in the ICUs of Alexandria hospitals [4].

Increased health care expenditure for patients infected with MDR strains is one of the burdens imposed by AMR [7]. The Organization for Economic Cooperation and Development (OECD) reported that resistant microbes currently cause 700,000 deaths annually. If the current trends persist, the number of deaths is expected to rise to ten million by 2050, displacing cancer as one of the prime causes of mortality [8].

To combat AMR, innovative approaches must be adopted in the research for novel antimicrobial medicines. As the use of synthetic chemicals to combat MDR strains is highly restricted because of health and environmental considerations [9], turning to natural products is, then, an excellent alternate to control the prevalence of AMR. *Centaurea spp* L. are well-known for their bioactive secondary metabolites with antimicrobial potential against Gram-positive, Gram-negative, and MDR bacteria [10–12]. They have been reported to possess medicinally important EOs and large numbers of terpenoids with more than 3000 different structures [12]. The antimicrobial activity of many *Centaurea spp.* L. (e.g., *C. pulcherrima*, *C. consanguinea*, *C*. *ptosomipappa*, *C. chamaerhaponticum*, *C. amanicola*, *C. sessilis*, *C. armena*, and *C*. *aladagensis*) has already received researchers’ attention [12]. *Centaurea pumilio*, Synonym *C. aegialophila* is a rare species that can be found scattered on sand dunes along the Egyptian Mediterranean coast [13]. Its dried root is frequently used as a fattening agent in traditional Egyptian medicine [14] and the indigenous people commonly use the peeled root to treat diabetes and bad breath. It has also been screened for efficacy against schistosomiasis and showed antioxidant activity [14]. *C. pumilio* is regarded as an endangered plant and has been included on the national Red List of the International Union for Conservation of Nature (IUCN) of threatened plants [13].

The objective of the present study was to extract EO and other active volatile constituents from the aerial part and the root of *C. pumilio* in order to assess the antimicrobial potential against 26 MDR strains collected from ICUs, 10 reference, and 16 clinical strains. To the best of the authors’ knowledge, the current work represents the first study to separate and assess the antimicrobial potential of the active constituents in *C. pumilio*.

## Methods

### Reagents and chemicals

The chemicals and reagents used in this study were purchased from Sigma-Aldrich (St. Louis, MO, USA).

### Plant materials

The plant was collected from its natural habitats on the Northern coast of Egypt during their flowering season (April and May 2018). It was identified as *Centaurea pumilio* L. by Prof. Dr. Salama El-Darer (Botany Department, Faculty of Science, Alexandria University, Egypt). A voucher specimen (CP019) was deposited in the herbarium of the Pharmacognosy Department. The fresh plant (Additional file 1) was air-dried, placed in a tightly sealed container, and stored in a cold, dark, dry place until the analyses were carried out. Permission to collect the plant samples was not required.

### Preparation of *C. pumilio* EO and extracts

**Fig. 1:**
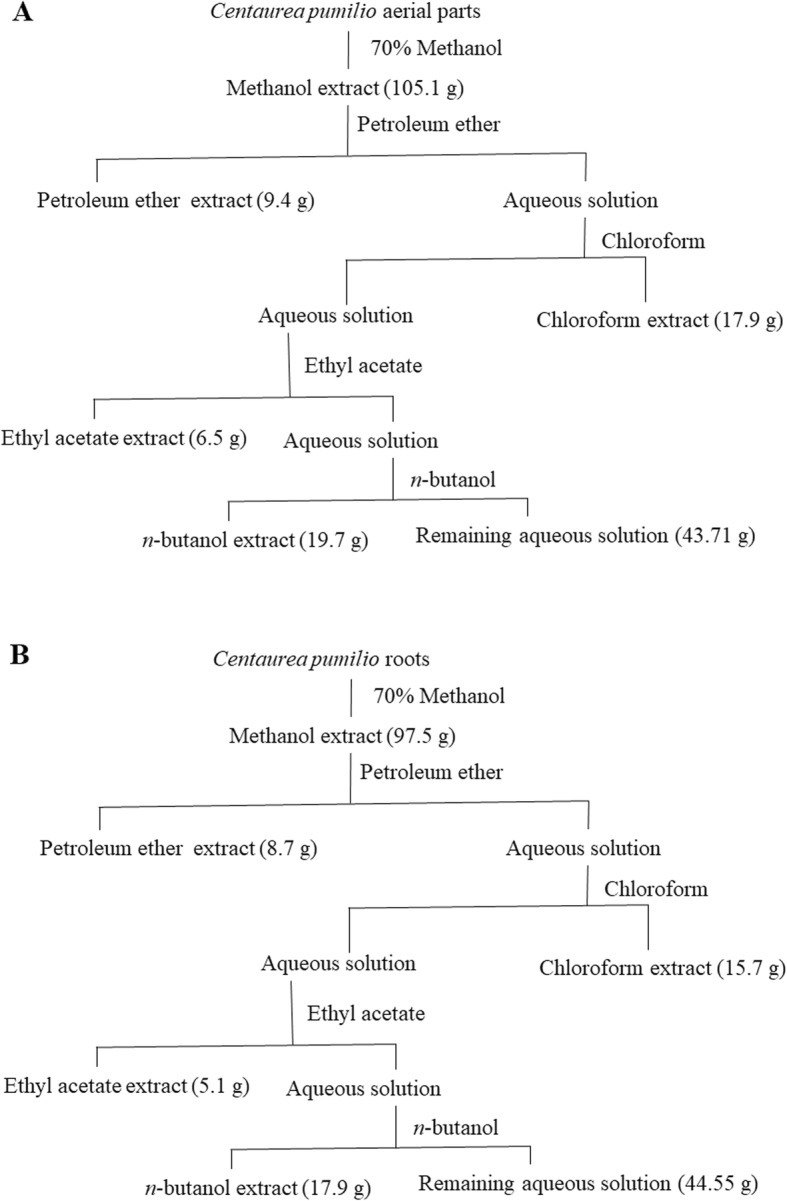
Yield of bioactive compounds extracted from *C. pumilio* by solvent-solvent extraction method. **a**: Aerial part. **b**: Root

The EO was extracted in accordance with previously reported procedures [15]. The air-dried aerial part (400 g) was subjected to hydrodistillation using a Clevenger apparatus. The resultant pale yellow oil (Additional file 2) was stored at − 20 °C. Meanwhile, a solvent/solvent extraction method was employed to extract the active constituents from the aerial part and the root [16]. The powdered air-dried aerial part and root of *C. pumilio* (700 g) were extracted separately with 4 L methanol (70%) until exhaustion. Both methanol extracts were filtered and concentrated under vacuum. The aqueous solutions were further extracted sequentially using four solvents with increasing polarities in the following order: petroleum ether, chloroform, ethyl acetate, and *n*-butanol. The organic phases of each extract from the aerial part and root, and the remaining aqueous extracts, were evaporated under reduced pressure (Fig. 1a, b).

### GC-MS analysis

The gas chromatography-mass spectrometry (GC-MS) analyses of *C. pumilio* EO and chloroform aerial part extract were carried out using a GC-MS instrument with the following specifications: a TRACE GC Ultra Gas Chromatographs (Thermo Scientific Corp., USA) coupled with a Thermo mass spectrometer detector (ISQ Single Quadrupole Mass Spectrometer) [15, 16]. The experimental conditions of the GC-MS system were as follows: TR-5MS capillary standard non-polar column, dimension: 30Mts, ID: 0.25 mm, and film thickness: 0.25 μm. The flow rate of the mobile phase (carrier gas: He) was set at 1.0 ml/min. In the gas chromatography phase, the temperature program (oven temperature) was set at 40 °C and then raised to 250 °C at 5 °C/min and the injection volume was 1 μL. The identification of the chemical constituents was de-convoluted using AMDIS software *(Available at http://**www.amdis.net**)* and by their retention indices (RI determined with reference to homologous series of n-alkanes C_9_–C_40_, under identical experimental conditions), MS library search (NIST 08 MS Library Version 2.0 f), and WILEY MS 9th Edition (Thermo Fisher Scientific Austria) and by comparing with the MS literature data. The relative amounts of individual components were calculated based on the GC peak area (FID response) without using a correction factor.

### Standard commercial antibiotics

Thirty-six commercial antibiotics (Sigma-Aldrich, St. Louis, MO, USA) were employed to assess the antibiotic resistance profile of the MDR strains. They were supplied as discs with known concentrations and are fully described in Additional file 3.

### Determination of antimicrobial activity

#### Bacterial strains

In the present study, ten reference strains: *Acinetobacter baumannii* ATCC 1797, *E. coli* ATCC 8739, *Enterococcus faecalis* ATCC 29212, *Enterobacter aerogenes* ATCC13048, *Klebseilla pneumonia* ATCC 700603, *Pseudomonas aeruginosa* ATCC 9027, *Proteus mirabilis* ATCC 14153, *Salmonella enterica* ATCC 14028, *Staphylococcus aureus* ATCC 6538, and *Candida albicans* ATCC 10231 were obtained from MIRCEN Faculty of Agriculture, Ain Shams University, Cairo, Egypt. Meanwhile, sixteen clinical strains were obtained from the Microbiology DepartmentFaculty of Medicine, Alexandria University, Egypt. Twenty-six multidrug resistant (MDR) strains were obtained from the ICUs Alexandria. The strains were identified using a three-step protocol: morphological identification, conventional biochemical tests and matrix-assisted laser desorption/ionization time of flight-mass spectrometry (MALDI-TOF-MS) (unpublished data). The sixteen clinical strains identified were *Enterococcus faecalis*, *Staphylococcus aureus*, *Bacillus cereus* (2 strains), *Streptococcus mutans*, *Bacillus pumilus*, *Escherichia coli*, *Pseudomonas aeruginosa*, *Acinetobacter baumannii*, *Salmonella enterica* serovar Typhi, *Stenotrophomonas maltophilia*, *Proteus mirabilis*, *Klebseilla pneumonia* (2 strains), *Enterobacter sp*. and *Candida albicans*. The twenty-six MDR strains were *S. aureus* MRSA (3 strains), *A. baumannii* (9 strains), *P. aeruginosa* (5 strains), *E. coli* (3 strains), *K. variicola* (one strain), and *K. pneumonia* (5 strains).

#### Cultivation conditions

The reference, clinical, and MDR strains were cultured on Müeller Hinton Broth (MHB, HiMedia, Mumbai, India) and Müeller Hinton Agar (MHA, HiMedia) at 37 °C for 18 h. However, *C. albicans* was cultivated on Sabouraud Dextrose Broth (SDB, HiMedia) and Sabouraud Dextrose Agar (SDA, HiMedia) at 30 °C for 18 h.

#### Disc diffusion assay

The antibiotic resistance patterns of the MDR strains and the antimicrobial potential of the EO and extracts from *C. pumilio* against all the strains tested (i.e., reference, clinical, and MDR strains) were determined using the Kirby-Bauer disc diffusion technique [17, 18] as described by CLSI guidelines. An inoculum of 0.5 McFarland was used to inoculate the MHA and SDA agar plates. The EO or plant extract (20 μL) was loaded onto the sterile filter paper discs at a concentration of 100 mg/ mL (2 mg/disc). Filter discs containing standard antimicrobial agents (ampicillin and fluconazole), EO, and extracts were employed in the pre-inoculated agar plates. The negative control plates were performed using dimethyl sulfoxide (DMSO). The diameters of the inhibition zones were measured and all the experiments were conducted in triplicate.

#### Determination of minimal inhibitory concentration

The minimum inhibitory concentrations (MIC) of the EO and extracts were determined with the broth micro-dilution method [18]. This was performed with 96-well microtiter plates using resazurin dye [19]. One hundred microliters (100 μL) of cell suspensions (5 × 10^5^ CFU/mL) were used to inoculate 50 μL of MHB containing different concentrations (15.62, 31.25, 62.5, 125, 250, 500, and 1000 μg/mL) of the EO or extract. The inoculated microtitre plates were incubated at 37 °C for 18 h. The MIC values were the lowest concentrations of the EO and plant extracts that suppressed visible growth of each strain tested in the microtitre plate. Experiments were conducted in triplicate.

### Statistical analysis

Data were statistically expressed in terms of means (*n* = 3) ± standard error (SE). Statistical analysis was run by the STATISTICA 10 of StatSoft, Inc. (2011) (Tulsa, Oklahoma, USA) [20]. Data variability was checked by one-way ANOVA at *P* < 0.05 for plant extracts and a standard antibiotic against strains (indicated with capital letters (A-E) in the same row). Factorial ANOVA was checked at *P* < 0.05 for each plant extract and the standard antibiotic to examine the factorial interaction between the reference and clinical strains and Gram-positive bacteria and Gram-negative bacteria (indicated with the small letters (a-c) in the same column). The data variability of the chloroform extract from the aerial part was checked by one-way ANOVA at *P* < 0.05 against the MDR strains; as indicated by the small letters (a-c) in the same column.

## Results

### Yield and chemical composition of EO and chloroform extracts

The yields of the extracts from the aerial part and the root after performing a solvent/solvent extraction are depicted in Fig. 1a and b. The chloroform extract and hydrodistillation of the aerial part yielded 2.56% (w/w) of brownish residue and 0.17% (v/w) of pale yellow EO, respectively. A TRACE GC Ultra Gas Chromatographs (Thermo Scientific Corp., USA) was used to identify and quantify these components. Eighteen volatile compounds were identified in the EO (Table 1) with sesquiterpene hydrocarbons representing the major class, including β-caryophyllene (29.33%) as the major volatile component, isogermacrene D (17.28%), *α-*cyperene (14.08%), butanoic acid-2-methyl, 2-methyl butyl (11.16%), caryophyllene oxide (10.49%), *α*-humulene (3.58%), *α*-copaene (2.14%), γ-elemene (1.24%), and T-muurolol (1.12%).

**Table 1:** Chemical composition of essential oil (EO) of *C. pumilio* analyzed by GC-Mass spectrometry

Peaks	Volatile compound	RI	LRI	Content (%)
1	butanoic acid-2-methyl,2-methyl butyl	1105	1106	11.16
2	Hexyl isovalerate	1251	1253	0.99
3	5-Methylhexyl 2-Methylbutanoate	1298	1299	0.72
4	*α*-copaene	1376	1375	2.14
5	*α*-Cyperene	1398	1398	14.08
6	β-Caryophyllene	1419	1419	29.33
7	*α*-Humulene	1448	1449	3.58
8	Isogermacrene D	1710	1708	17.28
9	γ-elemene	1449	1449	1.24
10	*α*-Muurolene	1492	1491	1.05
11	γ-Muurolene	1472	1473	0.98
12	Caryophyllene oxide	1571	1570	10.49
13	Caryophylladienol II	1631	1632	0.57
14	T-Muurolol	1628	1627	1.12
15	*α*-Valerenol	1737	1736	0.64
16	Germacra-4 (15),5,10 (14)-trien-1β-ol	1687	1686	3.86
17	Trans-Valerenyl isovalerate	2025	2024	0.43
18	Octacosane	444	442	0.32

*RI* retention index, *LRI* literature retention index

The GC-MS analysis of the chloroform extract showed eighteen compounds (Table 2). It contained ten major active compounds including hydrocarbons [pentadecane (17.83%), heptadecane (16.05%), hexadecane (8.89%), nonadecane (7.88%), heneicosane (7.30%), and heptacosane (6.08%)], long-chain alkanes (tetradecane, 9.65%), eicosane (7.10%), 3-Oxo-10(14)-epoxyguai-11(13)-en-6,12-olide (8.45%), cis-13-eicosenoic acid (5.64%), as well as other minor compounds. It is worth mentioning that this is, in fact, the first time a chemical analysis of the EO and the chloroform extract from the aerial part of *C. pumilio* has even been reported.

**Table 2:** Chemical analysis of chloroform aerial part extract of *C. pumilio* analyzed by GC-Mass spectrometry

Peaks	Volatile compound	Content (%)	M.W.	RI
1	n-tetradecane	9.65	198	1400
2	Carotene, 3,4-dihydro-1,1′,2,2′-tetra hydro-1′-hydroxy-1-methoxy-	0.58	584	1593
3	Pentadecane	17.83	212	1502
4	Lochneridine (Curan-17-oic acid,2,16-didehydro-20-hydroxy-19-oxo,methyl ester	0.67	398	2127
5	Hexadecane	8.89	226	1601
6	Heptadecane	16.05	240	1701
7	2a,4a-Epoxymethylphenanthrene-7-methanol,1,1-dimethyl-2-methoxy-8-(1,3-dithiin-2-ylidene) methyl-1,2,3,4,4a,4b,5,6,7,8,8a,9-dodecahydro-, acetate	0.73	490	1933
8	Hydrazinecarboxamide	0.45	75	829
9	7,8-Epoxylanostan-11-ol, 3-acetoxy	0.42	502	1734
10	Nonadecane	7.88	268	1900
11	Octadecamethyl cyclononasiloxane	0.63	666	1871
12	Eicosane	7.10	282	2000
13	Hexadecamethyl Cyclooctasiloxane	0.73	592	1700
14	Heneicosane	7.30	296	2100
15	Octasiloxane, 1,1,3,3,5,5,7,7,9,9,11,11,13,13,15,15-hexadecamethyl-	0.91	578	1759
16	Heptacosane	6.08	380	2700
17	Cis-13-eicosenoic acid	5.64	311	2365
18	3-Oxo-10 (14)-epoxyguai-11 (13)-en-6,12-olide	8.45	262	2010

*M.W.* molecular weight, *RI* retention index

### Antibiogram of MDR strains

The phenotypic profile of the 26 MDR strains against 36 commercial antibiotics is depicted in Additional file 3. There was a discrepancy in the antibiogram profile among the 26 MDR strains belonging either to the same species or different genera in terms of the number and group of the antibiotics. The uppermost MDR strains exhibited resistance against 10–16 antibiotics.

### Antimicrobial activity of *C. pumilio* extracts and EO against reference and clinical strains

The antimicrobial activities of *C. pumilio* EO and extracts against reference and clinical strains were assessed (Table 3). Using the factorial ANOVA at *P* < 0.05, the inhibition zone diameters (mm) of the chloroform, the ethyl acetate extracted from the aerial part, and the standard antibiotic showed significant data variability between the reference and clinical strains and between the Gram-positive and Gram-negative bacteria and their interactions (categorical factors). Statistical analysis proved that the highest significant data variability was observed between the Gram-positive and Gram-negative bacterial strains (*F* = 3.374, *P* < 0.01) compared to those of the reference and clinical strains (*F* = 3.744, *P* < 0.05) and their interactions (*F* = 2.176, *P* < 0.05). No significant data variability was observed for the 70% methanol extract from the aerial part, the chloroform root extract, or the EO. The chloroform extract from the aerial part showed the highest inhibition zone diameters compared to the standard antibiotic, other extracts, and the EO. The inhibition zone diameters of the chloroform aerial part extract varied significantly as indicated by the different letters between the reference (mean = 27.11 mm) and clinical strains (mean = 22.42 mm). It also showed significant variance between the Gram-negative (mean = 20.40 mm) and Gram-positive clinical bacterial strains (mean = 26.47 mm). Furthermore, it showed the highest significant variability inferred by one-way ANOVA at *P* < 0.05 among the other extracts and the standard antibiotic against each strain. The antibacterial activity of the ethyl acetate extract from the aerial part varied significantly between the reference (mean = 16.56 mm) and clinical strains (mean = 15.41 mm), and the Gram-negative (mean = 14.00 mm) and Gram-positive clinical bacterial strains (mean = 18.58 mm).

**Table 3:** Inhibition zone diameters (mm) of *C. pumilio* extracts and EO against reference and clinical strains

Stains	Inhibition zone diameter (mm)					
	70% methanol aerial part extract	Chloroform aerial part extract	Ethyl acetate aerial part extract	Chloroform root extract	EO	Standard antimicrobial agent
**Reference strains**						
*A. baumannii* ATCC 1797	10.00 ± 0.58^A^	25.00 ± 0.58^b;C^	16.00 ± 0.00^bc;B^	ND	ND	11.33 ± 0.33^a;A^
*E. coli* ATCC 8739	8.67 ± 0.33^A^	29.33 ± 0.33^b;D^	13.00 ± 0.58^bc;B^	10.00 ± 0.00^A^	9.33 ± 0.33^A^	20.00 ± 0.58^a;C^
*Ent. Faecalis* ATCC 29212	11.33 ± 0.33^B^	29.67 ± −0.33^b;D^	11.33 ± 0.33^bc;B^	8.33 ± 0.33^A^	8.33 ± 0.33^A^	19.00 ± 0.00^a;C^
*Enterobacter aerogenes* ATCC13048	7.67 ± 0.33^A^	17.67 ± 0.33^b;C^	9.67 ± 0.33^bc;B^	ND	ND	ND
*K. pneumonia* ATCC 700603	8.00 ± 0.00^A^	22.67 ± 0.33^b;C^	11.67 ± 0.33^bc;B^	8.67 ± 0.33^A^	10.67 ± 0.33^B^	ND
*P. aeruginosa* ATCC 9027	16.00 ± 0.00^C^	29.67 ± 0.33^b;E^	19.67 ± 0.33^bc;D^	11.00 ± 0.00^A^	14.67 ± 0.33^B^	ND
*P. mirabilis* ATCC 14153	8.67 ± 0.33^C^	29.33 ± 0.33^b;E^	15.67 ± 0.33^bc;D^	11.67 ± 00^A^	13.33 ± 0.33^B^	12.00 ± 0.00^a;AB^
*S. enterica* ATCC 14028	7.33 ± 0.33^A^	30.00 ± 0.00^b;D^	24.00 ± 0.00^bc;C^	7.67 ± 0.33^A^	12.33 ± 0.33^B^	ND
*S. aureus* ATCC 6538	12.76 ± 0.33^B^	30.67 ± 0.33^ab;E^	28.00 ± 0.00^c;D^	14.33 ± 0.33^A^	13.33 ± 0.33^A^	25.00 ± 0.00^bc;C^
*C. albicans* ATCC 10231	8.33 ± 0.33^A^	27.00 ± 0.58^abc;C^	ND	12.67 ± 0.33^B^	ND	30.00 ± 0.00^c;D^
**Clinical strains**						
*A. baumannii*	18.67 ± 0.33^A^	10.67 ± 0.33^ac;B^	17.67 ± 0.33^ab;A^	ND	ND	ND
*E. coli*	15.00 ± 0.58^B^	21.33 ± 0.33^ac;C^	19.33 ± 0.33^ab;C^	10.00 ± 0.58^A^	9.33 ± 0.33^A^	14.33 ± 0.03^ab;B^
*Ent. faecalis*	8.67 ± 0.67^B^	20.67 ± 0.33^ac;D^	14.33 ± 0.33^ab;C^	11.67 ± 0.33^A^	10.00 ± 0.58^A^	24.67 ± 0.33^ab;E^
*Enterobacter sp.*	15.67 ± 0.33^A^	16.67 ± 0.33^ac;A^	9.33 ± 0.33^ab;C^	ND	ND	8.00 ± 0.00^ab;B^
*K. pneumonia* strain1	8.33 ± 0.33^A^	23.67 ± 0.33^ac;D^	7.33 ± 0.33^ab;A^	8.33 ± 0.33^A^	11.33 ± 0.33^B^	19.00 ± 0.00^ab;C^
*K. pneumonia* strain2	15.00 ± 0.58^B^	25.00 ± 0.00^ac;D^	18.33 ± 0.33^ab;C^	10.67 ± 0.33^A^	11.67 ± 0.33^A^	15.33 ± 0.33^ab;B^
*P. aeruginosa*	11.00 ± 0.58^A^	24.00 ± 0.00^ac;C^	14.33 ± 0.33^ab;B^	11.67 ± 0.33^A^	13.67 ± 0.33^B^	11.00 ± 0.00^ab;A^
*P. mirabilis*	15.00 ± 0.58^A^	28.00 ± 0.00^ac;D^	17.00 ± 0.58^ab;A^	12.33 ± 0.33^C^	16.00 ± 0.58^A^	9.00 ± 0.00^ab;B^
*S. enterica*	14.00 ± 0.00^A^	25.00 ± 0.00^ac;E^	8.33 ± 0.33^ab;B^	15.33 ± 0.33^D^	13.67 ± 0.33^A^	11.33 ± 0.33^ab;C^
*Steno. maltophilia*	ND	9.00 ± 0.00^ac;A^	ND	13.33 ± 0.33^B^	ND	19.33 ± 0.33^ab;C^
*B. cereus* strain1	8.67 ± 0.67^A^	30.00 ± 0.00^ab;D^	17.00 ± 0.58^bc;C^	10.67 ± 0.33^B^	10.33 ± 0.33^AB^	16.33 ± 0.33^ab;C^
*B. cerues* strain2	14.00 ± 0.58^A^	28.00 ± 0.00^ab;D^	17.33 ± 0.33^bc;B^	12.00 ± 0.00^C^	13.67 ± 0.33^A^	16.33 ± 0.33^ab;B^
*B. pumilus*	13.33 ± 0.33^A^	30.33 ± 0.33^ab;D^	18.67 ± 0.33^bc;C^	12.33 ± 0.33^A^	12.67 ± 0.33^A^	7.00 ± 0.00^ab;B^
*S. aureus*	9.67 ± 0.88^AB^	27.67 ± 0.33^ab;E^	21.33 ± 0.33^bc;D^	9.00 ± 0.00^A^	10.67 ± 0.33^B^	13.00 ± 0.00^ab;C^
*Strep. mutans*	ND	16.33 ± 0.33^ab;B^	ND	10.33 ± 0.33^A^	ND	ND
*C. albicans*	7.33 ± 0.33^A^	12.00 ± 0.00^c;C^	ND	10.33 ± 0.33^B^	ND	20.00 ± 0.00^abc;D^

Different small letters (a-c) in the same column indicate significant data variability checked by factorial ANOVA at *P* < 0.05. No letters indicate no significant variability

Different capital letters (A-E) in the same row indicate significant data variability checked by one-way ANOVA at *P* < 0.05

*EO* essential oil, *ND* not detected

### Antimicrobial activity of *C. pumilio* extracts and EO against MDR strains

Of the four extracts studied, only the chloroform aerial part extract showed potent antimicrobial activity against all the MDR strains tested (Table 4). Using the one-way ANOVA, the chloroform aerial part extract showed significant data variability among the MDR strains tested (*F* = 3.606, *P* < 0.05), as indicated by the different letters (a-c). A discrepancy in the antimicrobial potential showed by the chloroform aerial part extract was noted among different strains of the same species. Among the nine MDR *A*. *baumannii* tested, inhibition zone diameters ranged from 11.33 ± 0.33 mm to 23.33 ± 0.33 mm. Whilst, among the five MDR tested *K. pneumonia*, inhibition zone diameters ranged from 12.00 ± 0.00 mm to 25.00 ± 0.00 mm. For the five MDR tested *P. aeruginosa* and the three MRSA strains tested, inhibition zone diameters ranged from 17.33 ± 0.33 mm to 27.00 ± 0.00 mm and 20.00 ± 0.00 mm to 28.00 ± 0.00 mm, respectively. Conversely, a negligible discrepancy in the range of the inhibition zone diameter was noted among the three MDR *E. coli* tested.

**Table 4:** Inhibition zone diameters of chloroform aerial part *C. pumilio* extract and EO against MDR strains

Inhibition zone diameter (mm)		
MDR Strain	Chloroform aerial part extract	EO
*A. baumannii* strain1	11.67 ± 0.33^abc^	–
*A. baumannii* strain2	22.00 ± 0.00^abc^	–
*A. baumannii* strain3	20.00 ± 0.00^abc^	–
*A. baumannii* strain4	16.00 ± 0.00^abc^	–
*A. baumannii* strain5	11.33 ± 0.33^abc^	–
*A. baumannii* strain6	21.67 ± 0.33^abc^	–
*A. baumannii* strain7	14.67 ± 0.33^abc^	–
*A. baumannii* strain8	20.00 ± 0.00^abc^	–
*A. baumannii* strain9	23.33 ± 0.33^abc^	–
*E. coil* strain1	14.67 ± 0.33^ab^	–
*E. coli* strain2	17.67 ± 0.33^ab^	9.00 ± 0.58
*E. coli* strain3	14.33 ± 0.33^ab^	–
*K. pneumonia* strain1	16.00 ± 0.00^abc^	–
*K. pneumonia* strain2	12.00 ± 0.00^abc^	–
*K. pneumonia* strain3	12.67 ± 0.33^abc^	–
*K. pneumonia* strain4	25.00 ± 0.00^abc^	–
*K. pneumonia* strain5	24.00 ± 0.00^abc^	12.67 ± 0.33
*K. variicola*	12.00 ± 0.00^a^	10.33 ± 0.33
*P. aeruginosa* strain1	20.00 ± 0.00^c^	–
*P. aeruginosa* strain2	25.00 ± 0.00^c^	7.33 ± 0.33
*P. aeruginosa* strain3	27.00 ± 0.00^c^	7.33 ± 0.33
*P. aeruginosa* strain4	17.33 ± 0.33^c^	9.33 ± 0.33
*P. aeruginosa* strain5	25.00 ± 0.00^c^	11.33 ± 0.33
*S. aureus* MRSA strain1	21.00 ± 0.00^bc^	4.00 ± 0.00
*S. aureus* MRSA strain2	20.00 ± 0.00^bc^	–
*S. aureus* MRSA strain3	28.00 ± 0.00^bc^	10.00 ± 0.58

Different small letters (a-c) indicate significant data variability at *P* < 0.05 checked by one-way ANOVA. No letters indicate no significant variability

*MDR* multidrug resistant, *EO* essential oil

The growth of only nine out of the twenty-six tested MDR strains was inhibited by the EO (Table 4). At most, the highest inhibition zone diameter was 12.67 ± 0.33 mm. The antimicrobial activity of the EO was not noticed against the nine MDR *A. baumannii* tested. EO inhibited the growth of only one out of the three MDR *E. coli* strains tested and only one out of five MDR *K. pneumonia* strains tested*.* In contrast, the growth of all the tested MDR *P. aeruginosa* strains was negatively influenced except for one strain. For the MRSA strains, only two out of three tested strains were adversely suppressed by EO.

### MIC of *C. pumilio* extracts and EO against reference and clinical strains

At most, the highest antimicrobial activity among the four extracts was evidenced by the lowest MIC values observed (Table 5). With regard to the lowest MIC values, the antimicrobial potential of the extracts was in the following order: chloroform aerial part extract >> > chloroform root extract > > methanol aerial part extract = ethyl acetate aerial part extract, when compared to MIC values of standard antibiotics. The chloroform aerial part extract exhibited an MIC value of 31.25 μg/mL against the Gram-negative bacteria, Gram-positive bacteria, and *C.albicans*. However, the chloroform root extract showed an MIC value of 62.5 μg/mL against the Gram-negative bacteria and Gram-positive bacteria. At most, the chloroform aerial part extract demonstrated a twofold increase in the antibacterial and anticandidal activity as compared to the standard antibiotic against the clinical strains of *K. pneumonia*, *P. aeruginosa*, *P. mirabilis*, *S. enetrica*, and *C. albicans*. Moreover, a four-fold increase in the antibacterial activity of the chloroform aerial part extract was noted against *B. pumilus*. In contrast, the MIC values of 125–250 μg/mL of the methanol and ethyl acetate aerial part extracts were noticed against Gram-negative bacteria and Gram-positive bacteria.

**Table 5:** Minimum inhibitory concentration (MIC) of *C. pumilio* extracts and EO against reference and clinical strains

Strains	MIC (μg/mL)					
	70% Methanol aerial part extract	Chloroform aerial part extract	Ethyl Acetate aerial part	Chloroform root extract	EO	Standard antimicrobial agent
**Reference strains**						
*A. baumannii* ATCC 1797	125.0	62.50	125	ND	ND	62.50
*E. coli* ATCC 8739	125.0	31.25	125	250.0	62.50	7.80
*Ent. faecalis* ATCC 29212	125.0	62.50	250	62.5	62.50	0.97
*E. aerogenes* ATCC 13048	125.0	62.50	125	ND	ND	ND
*K. pneumonia* ATCC 700603	250.0	62.50	125	250.0	125.00	ND
*P. aeruginosa* ATCC 9027	250.0	62.50	125	125.0	62.50	ND
*Prot. mirabilis* ATCC 14153	250.0	62.50	250	125.0	62.50	15.62
*S. enterica* ATCC 14028	125.0	62.50	125	125.0	31.25	ND
*S. aureus* ATCC 6538	125.0	31.25	250	62.5	62.50	1.95
*C. albicans* ATCC 10231	62.5	62.50	ND	250.0	ND	1.95
**Clinical strains**						
*A. baumannii*	250.0	125.00	250	ND	ND	ND
*E. coli*	250.0	62.50	250	125.0	125.00	3.90
*Ent. faecalis*	250.0	31.25	125	125.0	125.00	7.81
*Enterobacter sp.*	250.0	125.00	250	ND	ND	125.00
*K. pneumonia* strain 1	125.0	62.50	250	125.0	125.00	31.25
*K. pneumonia* strain 2	125.0	31.25	250	125.0	62.50	62.50
*P. aeruginosa*	125.0	31.25	250	62.5	125.00	125.00
*Prot. mirabilis*	125.0	31.25	125	62.5	125.00	62.50
*S. enterica*	250.0	31.25	250	62.5	62.50	125.00
*Steno. maltophilia*	125.0	125.00	ND	125.0	ND	125.00
*B. cereus* strain 1	125.0	62.50	125	125.0	125.00	7.81
*B. cerues* strain 2	250.0	62.50	125	125.0	125.00	31.25
*B. pumilus*	125.0	31.25	250	62.5.0	125.00	125.00
*S. aureus*	62.5	62.50	125	125.0	31.25	31.25
*Strep. mutans*	ND	62.50	ND	62.5	ND	ND
*C. albicans*	125.0	31.25	ND	125.0	ND	62.50

*EO* essential oil, *ND* not detected

The common MIC values for the EO were 62.50 μg/mL and 125 μg/mL for reference and clinical strains, respectively, against the Gram-negative bacteria and Gram-positive bacteria (Table 5).

### MIC of *C. pumilio* chloroform aerial part extract and EO against MDR strains

The chloroform aerial part extract revealed its strong broad-spectrum antibacterial activity against the MDR strains with MIC values of 15.62 μg/mL (against 2 MDR strains), 31.25 μg/mL (against 7 MDR strains), 62.5 μg/mL (against 14 MDR strains), 125 μg/mL (against 2 MDR strains), and 250 (against one MDR strain). Moreover, these MIC values varied greatly among the strains belonging to the same species and among the strains of different species (Table 6). For instance, among the nine tested MDR *A. baumannii*, four MIC values of the *C. pumilio* chloroform aerial part extract were observed: 31.25, 62.5, 125, and 250 μg/mL. For the MIC values of the *C. pumilio* chloroform aerial part extract against MDR *K. pneumonia*, three values were noted*:* 15.62, 31.25, and 62.5 μg/mL, and these very same values were obtained against MDR *P. aeruginosa* as well. Conversely, only 62.5 and 125 μg/mL MIC values were noted against MRSA.

**Table 6:** Minimum inhibitory concentration (MIC) of *C. pumilio* chloroform extract and essential oil (EO) against 26 MDR strains

MDR strains	MIC (μg/mL)	
	Chloroform aerial part extract	EO
*A. baumannii* strain1	31.25	–
*A. baumannii* strain2	250.00	–
*A. baumannii* strain3	62.50	–
*A. baumannii* strain4	62.50	–
*A. baumannii* strain5	125.00	–
*A. baumannii* strain6	31.25	–
*A. baumannii* strain7	62.50	–
*A. baumannii* strain8	62.50	–
*A. baumannii* strain9	31.25	–
*E. coli* strain1	31.25	–
*E. coli* strain2	62.50	62.50
*E. coli* strain3	62.50	–
*K. pneumonia* strain1	15.62	–
*K. pneumonia* strain2	62.50	–
*K. pneumonia* strain3	62.50	–
*K. pneumonia* strain4	62.50	–
*K. pneumonia* strain5	31.25	–
*K. variicola*	62.50	125.00
*P. aeruginosa* strain1	62.50	–
*P. aeruginosa* strain2	62.50	62.50
*P. aeruginosa* strain3	15.62	62.50
*P. aeruginosa* strain4	31.25	62.50
*P. aeruginosa* strain5	31.25	62.50
*S. aureus* MRSA strain1	125.00	250.00
*S. aureus* MRSA strain2	62.50	–
*S. aureus* MRSA strain3	62.50	250.00

*MDR* multidrug resistant

With regard to the EO MIC values, one fixed MIC value of 62.5 μg/mL was noted against MDR *P. aeruginosa*. However, two distal MIC values of 62.5 and 250 μg/mL were recorded against MRSA strains (Table 6). It showed antimicrobial activity against MDR *E. coli* and *K. variicola* with MIC values of 62.5 and 125 μg/mL, respectively.

## Discussion

In the ongoing search for natural antibiotics to treat human MDR infections, the current work focuses on looking for natural antimicrobial agents from *C. pumilio* extracts and EO.

Although the extracts and EOs of the genus *Centaurea* have been extensively studied and have showed remarkable antimicrobial activity against susceptible and MDR strains [11, 12, 21], the antimicrobial activity of *C. pumilio* has not been well explored.

The EO from *C. pumilio* inhibited *S. aureus* (MIC, 31.52 μg/mL), which is in accordance with *C. carthamoides* EO [12], and it also showed much higher antimicrobial activity against *S. aureus*, *S. enetrica*, and *E. coli* (with MIC values of 31.25, 62.50, and 125 μg/mL, respectively), when compared to those of *C. chamaer haponticum* EO [12]. β-caryophyllene, a natural sesquiterpene, has been reported for its strong antimicrobial activity [22] and has previously been detected in the EOs of C*. aladaghensis*, *C. amanicola*, *C. appendicigera*, *C. cheirolepidoides*, *C. deflexa*, *C. lanigera*, and *C. mucronifera* [21]. It is worth mentioning that *C. pumilio* EO has the second-highest percentage of β-caryophyllene (29.33%) after *C. deflexa* (33.9%) [21]. The EO from *C. pumilio* demonstrated higher antimicrobial activity as indicated by the MIC values of 125, 125, and 62.5 μg/mL against *B. cereus*, *P. aeruginosa*, and *K. pneumonia*, respectively, when compared to those of *C. solstitialis* [15], *C. appendicigera*, and *C. helenioides* EOs [23]. *C. pumilio* EO also displayed more potent antibacterial activity than *C. aladagensis* [24], *C. lycopifolia*, and *C. cheirolopha* EOs [25]. Isogermacrene D, a monocyclic sesquiterpene hydrocarbon, has previously been reported in the EOs of *C. antiochia, C. ptosimopappoides, C. babylonica, C. antitauri*, *C. balsamita*, *C. cheirolepidoides*, and *C. aladaghensis* [21] and the EOs from *C. helenioides* [23], *C. rupestris* [18], *C. solstitialis* [15], *C. baseri* [26], *C. cinerari*, and *C. napifolia* [27] have been noted for their isogermacrene D content and antimicrobial activity that match with the present findings of *C. pumilio* EO.

The current study deals with the antimicrobial potential of four extracts; methanol, chloroform, and ethyl acetate aerial part extract and chloroform root extract. The methanol extract demonstrated high antibacterial activity against *S. aureus* and *A. baumannii* strains (MIC, 62.50 μg/mL and 250 μg/mL, respectively), whereas in comparison, *C. ragusina* methanol extract shows less antibacterial activity [11]. Additionally, it exhibited the highest antimicrobial potential against *S. aureus*, *B. cerues*, and *E. coli* with MIC of 62.50, 125, and 250 μg/mL, respectively, when compared to the same extract from twelve *Centaurea spp*. [28]. *C. persica, C. polyclada,* and *C. consanguinea* methanol extracts also demonstrated anticandidal activity with MIC of 125 μg/mL [29].

Neither the *C. pumilio* methanol nor the ethyl acetate extract showed any activity against *Strep. mutans,* which is in agreement with *C. austro-anatolica* [17] and *C. cariensis* [30]. This discrepancy might be because of species differences. Unlike the *C. cadmea* methanol root extract [31] and *C. montana* root ethyl acetate and *n*-butanol extracts, *C. pumilio* chloroform root extract did show antimicrobial activity [32], and contrary to the *C. montana* root chloroform extract [32], the corresponding *C. pumilio* extract did suppress *C. albicans* growth (MIC, 125 μg/mL).

Interestingly, the *C. pumilio* chloroform aerial part extract exhibited a twofold increase in the anticandidal activity to that of the standard antibiotic. This anticandidal activity is also seen in *C. thessala* and *C. attica* chloroform aerial part extracts [33], but not in the chloroform extracts of *C. austro-anatolica*, *C. cariensis* subsp*. niveo-tomentosa,* and *C. ensiformis* [17, 30, 34]. *Strep. mutans* and *Steno. maltophilia* were inhibited by chloroform extracts of both the root and aerial part of *C. pumilio* (MIC of 62.50 and 125 μg/mL, respectively). Meanwhile, the *C. pumilio* chloroform aerial part extract exhibited higher antibacterial activity against MRSA (21, 20, and 28 mm) than that of *C. austro-anatolica* [17] and *C. cariensis* [30]. Previous studies demonstrated that the chloroform extracts from the aerial part of *C. austro-anatolica* and *C. cariensis* had antibacterial activity against MDR *Steno. maltophilia* strains [17, 30].

The *C. pumilio* EO inhibited the growth of *Ent. faecalis* (MIC, 125 μg/mL) more than the EO from *C. helenioides* did [23] and MRSA (MIC, 250 μg/mL) more than the *C. baseri* EO [26]. The antibacterial activity of the *C. pumilio* EO against MRSA may be attributed to its 15% isogermacrene D content [12]. The antimicrobial activity of the *C. pumilio* EO against the reference, clinical, and MDR strains might be attributed to its high content of sesquiterpenes [12]: β-caryophyllene (29.33%), isogermacrene D (17.28%), *α*-cyperene (14.08%), and caryophyllene oxide (10.49%). The literature reviews revealed that EOs containing high percentages of β-caryophyllene augmented antibiotic potency against Gram-negative bacteria [35]. This would suggest that *C. pumilio* EO is a good candidate for new formulations that can contribute to reducing AMR [35]. The current study revealed for the first time the presence of butanoic acid, 2-methyl-, 2-methylbutyl ester which has been reported for its antimicrobial activity as it is the main component in *Ammi visnaga* L. EO [36]. Therefore, it might also contribute to the antimicrobial activity of the *C. pumilio* EO. So far, the findings of the current study concerning the antimicrobial activity of the *C. pumilio* EO against the MDR *P. aeruginosa*, *E. coli*, and *K. variicola* strains can be considered as the first report among the *Centaurea* genus. This would underpin the great potential the *C. pumilio* EO has in the fight against the MDR strains with global health implications the WHO and CDC annual statistical estimates have reported [2]. As a consequence, it represents an excellent natural alternative to the inefficient synthetic anti-MDR agents.

The GC-MS analysis of the *C. pumilio* chloroform aerial part extract reported hydrocarbons as the most abundant constituents and showed antibacterial activity against the clinical and MDR pathogens [37–41]. Pentadecane and heptadecane are the major hydrocarbons and these have been reported for their antimicrobial activity [39–41]. Tetradecane, hexadecane, nonadecane, and heneicosane were recorded as antimicrobial agents [41]. Eicosane, a long-chain fatty acid, has been reported for its antibacterial activity [41]. Pentadecane, tetradecane, hexadecane, nonadecane, heneicosane, eicosane, and heptacosane have previously been detected in *C. napifoli*a, *C. iconiensis*, *C. antiochia, C. aladaghensis*, *C. lanigera*, *C. iconiensis*, and *C. solstitialis*, respectively [15, 21, 27]. The anticandidal activity of the *C. pumilio* chloroform aerial part extract might be accredited to 3-oxo-10(14)-epoxyguai-11(13)-en-6,12-olide and curan-17-oic acid,2,16-didehydro-20-hydroxy-19-oxo,methylester which have been described as antifungal agents [41, 42]. In addition, Cis-13-eicosenoic acid was detected in *Camilla sinesis* extract that was described for its antimicrobial activity against MRSA and MDR *P. aeureginosa* with MIC of 400 μg/mL and 800 μg/mL, respectively [43]. Consequently, the significant antimicrobial activity of the *C. pumilio* chloroform aerial part extract against the MDR *P. aeureginosa* and MRSA strains (MIC of 62.5 and 15.62 μg/mL, respectively) may be attributed to its content of cis-13-eicosenoic acid. The antimicrobial activities of the *C. pumilio* chloroform aerial part extract against the reference, clinical, and MDR pathogens are likely attributed to these bioactive compounds. The presence of these highly synergistic active compounds with high percentages in the chloroform aerial part extract may illustrate its potent antimicrobial activity against susceptible and MDR bacteria [44]. Noticeably, it is the aerial part of *C. pumilio* that contains more antimicrobial compounds in the EO, methanol, ethyl acetate, and chloroform extract compared to those in the root.

## Conclusions

The current study is considered the first attempt to extract and assess the antimicrobial activity of the EO and extracts from *C. pumilio* not only against susceptible bacteria, but also MDR strains. This study does support the usage of *C. pumilio* as a medicinal plant as it is an extremely rich supplier of potent broad-spectrum antimicrobial bioactive compounds. The results of the research on the antimicrobial activity of *C. pumilio* showed that the extracts from the plant do exhibit strong activity, which is particularly promising given that antimicrobial resistance has become a major health issue worldwide. There is clear potential for *C. pumilio* EO and extracts to be exploited in the pharmaceutical industries and in the formulations of food additives for prophylaxis purposes. That said, further studies are mandatory to purify the chemical constituents of the antimicrobial fractions to better illustrate their mode of action. Moreover, the cytotoxicity levels of *C. pumilio* EO and extracts should also be studied further in vitro and in vivo to develop stable drugs for human/animal use.

## Supplementary information

**Additional file 1: **Morphology of the wild rare plant *C. pumilio* L..

**Additional file 2: **Extracted essential oil (EO) from the wild rare plant *C. pumilio* L..

**Additional file 3: Table S1.** Profile of antibiotic resistance of 26 MDR strains from ICUs in Alexandria hospitals.

## Data Availability

The authors declare that all data are included in the manuscript without any restriction.

## Abbreviations

AMR: Antimicrobial resistance
CDC: Centre for disease control and prevention
DMSO: Dimethyl sulfoxide
EO: Essential oil
GC-MS: Gas chromatography-mass spectrometry
ICUs: Intensive care units
IUCN: International union for conservation of nature
MALDI-TOF-MS: Matrix-assisted laser desorption/ionization time of flight-mass spectrometry
MDR: Multidrug resistant
MIC: Minimum inhibitory concentration
OECD: Organization for economic cooperation and development
