# Supplementary figures and images for: Antibacterial activity of Centaurea pumilio L. root and aerial part extracts against some multidrug resistant bacteria
Source: BMC Complement Med Ther. 2020 Mar 12;20:79. doi: 10.1186/s12906-020-2876-y (PMC7076891; doi:10.1186/s12906-020-2876-y)

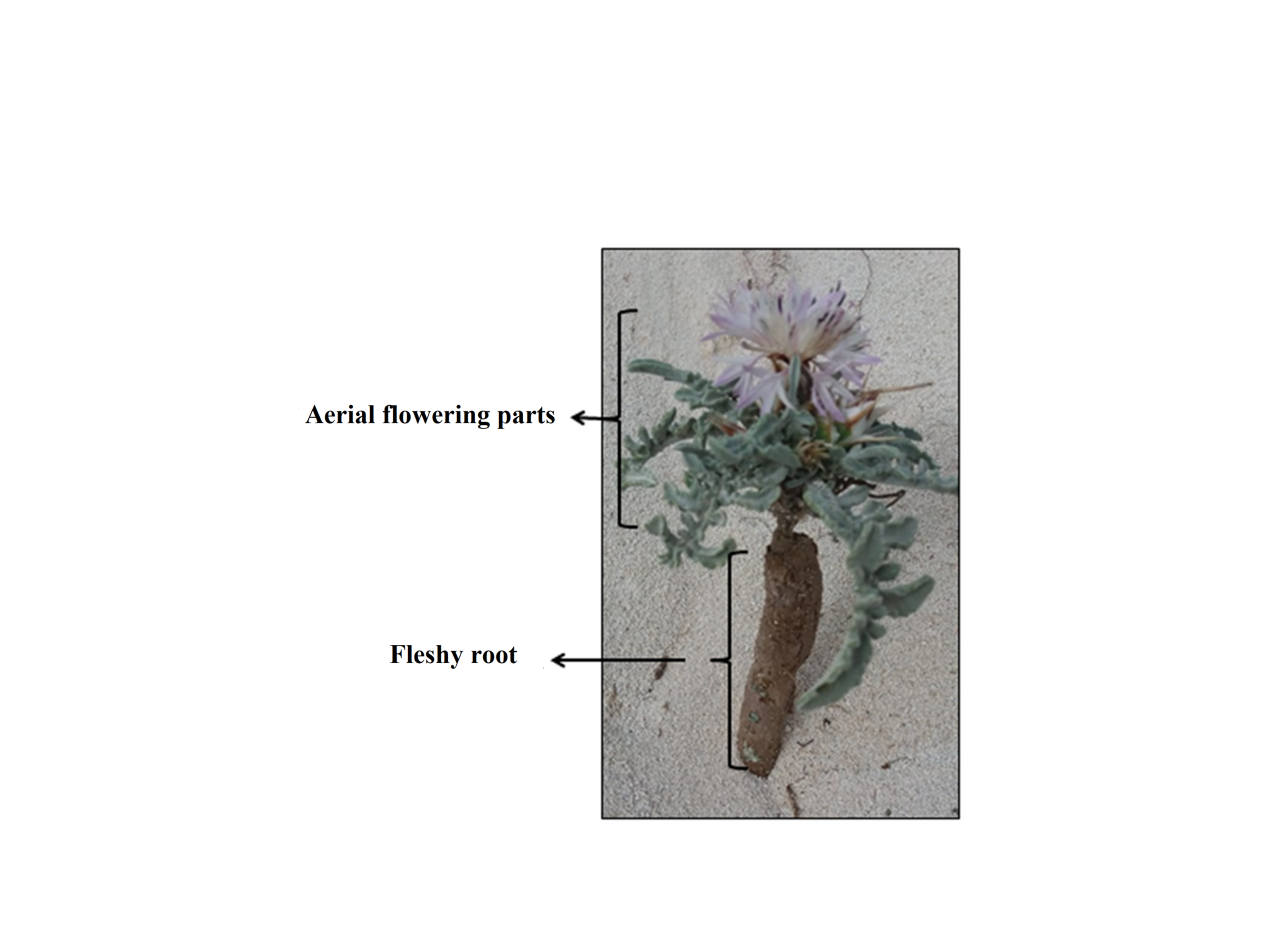

Supplement: Supplementary file 1 — Additional file 1: Morphology of the wild rare plant C. pumilio L.. [file 12906_2020_2876_MOESM1_ESM.png]

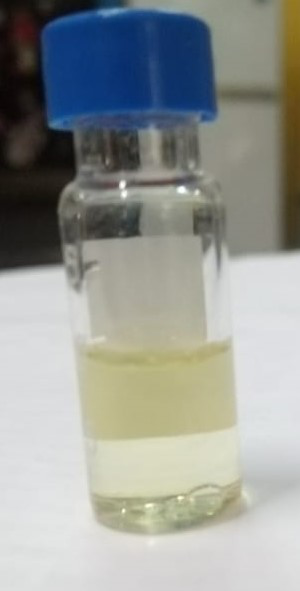

Supplement: Supplementary file 2 — Additional file 2: Extracted essential oil (EO) from the wild rare plant C. pumilio L.. [file 12906_2020_2876_MOESM2_ESM.png]
