# Supplementary material for: Antibacterial activity of Centaurea pumilio L. root and aerial part extracts against some multidrug resistant bacteria
Source: BMC Complement Med Ther. 2020 Mar 12;20:79. doi: 10.1186/s12906-020-2876-y (PMC7076891; doi:10.1186/s12906-020-2876-y)
Supplement: Supplementary file 3 — Additional file 3: Table S1. Profile of antibiotic resistance of 26 MDR strains from ICUs in Alexandria hospitals. [file 12906_2020_2876_MOESM3_ESM.docx]

**Table S1 Profile of antibiotic resistance of 26 MDR strains from ICUs in Alexandria hospitals.**

| **Isolates** | **Resistance profile** | |
| --- | --- | --- |
| *Acinetobacter baumannii* (strain1) | AMK, MEM, CTX, TOB, SXT, DOX, CST | |
| *A.baumainnii* (strain2) | | AMK, IPM, FEP, MEM, CAZ, CTX, GEN, DOX, CRO, LVX, TZP |
| *A. baumannii* (strain3) | | FEP, MEM, CAZ, GEN, DOX, CRO, LVX, AMK, |
| *A. baumannii* (strain4) | IPM, ETP, TOB, CXM, DOX, GEN, CFP, TZP, SXT | |
| *A. baumannii* (strain5) | | AMK, IPM, FEP, ATM, MPM, TOB, CAZ, GEN, CIP, TZP |
| *A.baumannii* (strain6) | | IPM, AMK, FEP, MEM, CTX, CAZ, CRO, CIP, SAM, CFP\SUL, , TZP, SXT |
| *A. baumannii* (strain7) | | AMK, IPM, FEP, GEN, CRO, CIP, SAM, TZP, SXT |
| *A. baumannii* (strain8) | | AMK, FEP, CIP, SAM, TZP, SXT |
| *A. baumannii* (strain9) | | AMK, CTX, GEN, LVX, SXT |
| *Escherichia coli* (strain1) | | IPM, CXM, TZP, NOR |
| *E.coli* (strain2) | | AMK, MEM, CTX, TZP, CIP |
| *E. coli* (strain3) | | IPM, MEM, ATM, FOX, CXM, CTX, GEN, CIP, TZP |
| *Klebseilla pneumonia* (strain1) | | AMK, IPM, FEP, FOX, MEM, ATM, CAZ, CTX, GEN, CRO, DOX, LVX, SAM, CFP\SUL, TZP, SXT |
| *K.pneumonia* (strain2) | | FEP, IPM, MEM, FOX, AMP, CTX, CAZ, CRO, TGC, SAM, CFP/SUL, , TZP, AMK, DOX |
| *K.pneumonia* (strain3) | | AMP, CTX, CRO, TGC, SXT, GEN, CFP, CIP |
| *K.pneumonia* (strain4) | | SAM, CXM, CTX, AMK, IPM, LVX, SXT, CIP, GEN |
| *K.pneumonia* (strain5) | | AMX, ERY, CTX, CFZ, FOX, ATM, CTX, TZP, SXT, CST, ETP, CIP |
| *K. variicola* | | AMP, SAM, CST |
| *Pseudomonas aeruginoa* (strain1) | | AMK, IPM, FEP, MPM, CAZ, CTX, TOB, GEN, CRO, CIP, SAM, TZP, SXT |
| *P. aeruginosa* (strain2) | | IPM, MEM, TOB, GEN, PIP, CIP, TZP |
| *P. aeruginosa* (strain3) | | AMK, IPM, FEP, MEM, ATM, TOB, CAZ, CIP, TZP |
| *P. aeruginosa* (strain4) | | AMK, IPM, FEP, TOB, CAZ, CIP, PIP, NOR |
| *P. aeruginosa* (strain5) | | ATM, PIP, CAZ, MEM, CIP, CTX, MPM, IPM, TZP |
| *Staphylococcus aureus* (MRSA1) | | FOX, PEN, CLI, GEN, TET, AZM,CIP, SXT, ERY |
| *St. aureus* (MRSA2) | | AMK, FOX, PEN, DOX, CFP/SUL |
| *St. aureus (*MRSA3) | | FOX, PEN, DOX, TZP |

MDR: multidrug resistant; ICU: intensive care unit; AMK: amikacin (30 µg); AMX: amoxicillin (20 µg); SAM: ampicillin-sulbactam (10/10 µg); AZM: azithromycin (15 µg); ATM: azetreonam (30 µg); FEP: cefepime (30 µg); CFZ: cefazolin (30 µg); CFP: cefoperazone (75 µg); CTX: cefotaxime (30 µg); FOX: cefoxitin (30 µg); CAZ: ceftazidime (30 µg); CRO: ceftriaxone (30 µg); CXM: cefuroxime (30 µg); CIP: ciprofloxacin (5 µg); CLI: clindamycin (2 µg); CST: colistin (10 µg); DOX: doxycycline (30 µg); ETP: ertapenem (10 µg); ERY: erythromycin (15 µg); GEN: gentamicin (10 µg ); IPM: imipenem (10 µg); LVX: levofloxacin (5 µg); MEM: meropenem (10 µg); NOR: norfloxacin (10 µg); OFX: ofloxacin (5 µg); PEN: penicillin (10 units); PIP: piperacillin (100 µg); TZP: piperacillin-tazobactam (100/10 µg); TET: tetracycline (30 µg); TGC: tigecycline (15 µg); TOB: tobramycin (10 µg); SXT: trimethoprim-sulfamethoxazole (1.25/23.75 µg); CHL: chloramphenicol (30µg); AMP: ampicillin (10 µg); CFP/SUL: cefoperazone/sulbactam (75/10 µg).
